# The Dynamics and Neural Correlates of Audio-Visual Integration Capacity as Determined by Temporal Unpredictability, Proactive Interference, and SOA

**DOI:** 10.1371/journal.pone.0168304

**Published:** 2016-12-15

**Authors:** Jonathan M. P. Wilbiks, Benjamin J. Dyson

**Affiliations:** 1 Department of Psychology, Ryerson University, Toronto, Ontario, Canada; 2 Department of Psychology, Mount Allison University, Sackville, New Brunswick, Canada; 3 School of Psychology, University of Sussex, Falmer, United Kingdom; Centre de neuroscience cognitive, FRANCE

## Abstract

Over 5 experiments, we challenge the idea that the capacity of audio-visual integration need be fixed at 1 item. We observe that the conditions under which audio-visual integration is most likely to exceed 1 occur when stimulus change operates at a slow rather than fast rate of presentation and when the task is of intermediate difficulty such as when low levels of proactive interference (3 rather than 8 interfering visual presentations) are combined with the temporal unpredictability of the critical frame (Experiment 2), or, high levels of proactive interference are combined with the temporal predictability of the critical frame (Experiment 4). Neural data suggest that capacity might also be determined by the quality of perceptual information entering working memory. Experiment 5 supported the proposition that audio-visual integration was at play during the previous experiments. The data are consistent with the dynamic nature usually associated with cross-modal binding, and while audio-visual integration capacity likely cannot exceed uni-modal capacity estimates, performance may be better than being able to associate only one visual stimulus with one auditory stimulus.

## Introduction

Capacity limits represent fundamental constraints of information processing [1,2]. One well-established limit refers to the number of objects that can be held concurrently in visual short-term memory (VSTM), with a variety of behavioural and electrophysiological evidence (e.g., [3]—[5]) points to an upper limit of 3 to 4 visual objects. Capacity limits have also been studied in other domains, notably, audio-visual integration. Relative to the capacity of VSTM, Van der Burg, Awh, & Olivers provide evidence for a “stricter, intersensory limitation” ([1], p. 350) regarding audio-visual integration in that only one visual event can be bound to any one sound. Using a variant on their ‘pip-and-pop’ paradigm (e.g., [6]), participants viewed a number of locations around an implied circle. The color of each location could change polarity (white to black, or vice versa) at each presentation and the number of locations that could change polarity was fixed across a trial (ranging from 1 to 8). After a variable number of non-critical frames in which a high degree of apparent motion is experienced, the critical frame is presented whereby an auditory tone is presented simultaneously with one such presentation. After further polarity change and a period of retention, participants are probed with one location from the array and asked whether that particular location changed polarity when the auditory tone was played. Proportion correct data showed that performance deteriorated as the number of changing locations increased and, importantly, audio-visual integration never exceeded 1 (estimated using *K*; [3]).

The suggestion of an upper bound for audio-visual integration is consistent with the idea of VSTM limits, but somewhat at odds with the reality of individual differences and the range of values often reported for VSTM capacity (1.5–*6* reported by [7]) and, indeed, AV capacity (0.70–*1*.*56* reported by [1] in their Experiment 1c [200 ms SOA] condition, or, 0.30–*1*.*36* in their Experiment 2 [150 ms SOA] condition). The data are also at odds with the multi-modal integration literature in general, which tends to emphasize its dynamic nature (e.g., [8], [9], [10]). It seemed possible to us that the factors that influence capacity in the visual domain such as perceptual load [11] and rate of presentation [12] should also modulate capacity in audio-visual integration. Firstly, a central tenet of perceptual load theory states that attention can be deployed to both target and distractor information under conditions of low rather than high load [11], with set size serving as a well-known operationalization of perceptual load. Given the temporal unpredictability of the critical trial where relevant locations can quickly become irrelevant (and vice versa), we believed the wide attentional lens provided by low perceptual load could well improve performance and lead to an increase in capacity. In their Experiment 1b Van der Burg et al. [1] noted that decreasing set size from 24 to 16 items led to a non-significant increase in audio-visual capacity. With a reported *p* > .1 using 9 participants, it is likely that the importance of set size has been underestimated. Secondly, the number of locations or objects that can be reliably tracked is in part determined by the rate of stimulus presentation. For example, Holcombe and Chen presented participants with two or three rings, each of which contained squares that rotated about a fixation point [13]. On different trials, one, two, or three of the squares were targets, and the rest were designated as distractors. Participants were asked to track the targets as they rotated, and then indicate which squares were target(s) at the end of a trial. The researchers found that with a single target, the temporal frequency limit was 143 ms, for two objects the limit was 250 ms, and, for three objects it was 385 ms. So it is likely that this temporal frequency limit makes it impossible for the capacity of integration to exceed one item at stimulus onset-asynchronies (SOAs) below 250 ms, and this aligns well with the finding of a capacity upper-bound of 1 using 150 or 200 ms, particularly with the high degree of apparent motion implied by the current paradigm. Van der Burg et al. [1] also noted that decreasing the speed of presentation between successive frames (SOA) from 150 to 200 ms led to a significant increase in performance, assumedly due a reduction in the number of incorrect audio-visual bindings ([1], p. 348). Given that perceptual load and SOA are both continuous variables, there seems good reason to expect that with more distinct manipulations, the capacity of audio-visual integration would exceed 1.

In the current experimental series, we put into clearer focus the factors that influence the capacity of audio-visual integration. We expect that the overall reduction of visual perceptual load from 16 or 24 items to 8 will result in the potential for increasing the capacity of audio-visual integration to be greater than one item. Additionally, we expect that including a slower SOA of 700 ms will provide potential similar opportunity for audio-visual integration capacity to increase. We also identified two additional factors within the paradigm that might influence capacity limits. First, there is a high degree of proactive interference in the original study, in that a large number of non-critical frames always preceded the critical frame in which an auditory tone was paired with a specific display. We hypothesised that reducing the amount of proactive interference by reducing the number of non-critical frames leading up to the critical one, would further enhance audio-visual capacity. Second, the temporal position of the critical frame could be situated at a number of different points within the trial. This temporal unpredictability would likely compromise capacity estimates and we additionally hypothesised that increasing the temporal predictability of the critical frame would facilitate an increased capacity of audio-visual integration.

In Experiment 1, we run a modified version of [1] examining AV capacity under both fast (200 ms) and slow (700 ms) SOA conditions with reduced perceptual load of 8 items, showing AV capacity to be no greater than 1. We further manipulated two critical experimental features: the degree of proactive interference generated by non-critical frames and the temporal predictability of the critical frame. In Experiment 1, as in [1], trials were presented with a relatively low level of temporal predictability, and a high degree of proactive interference. In Experiment 2, when the degree of proactive interference was reduced but the critical frame was still temporally unpredictable, AV capacity was shown to exceed 1 under conditions of slow SOA. In Experiment 3, we also held the critical frame constant with the prediction that this would further improve AV capacity but this was not found to be the case. We completed the series in Experiment 4 by investigating AV capacity under conditions of temporal predictability and high levels of proactive interference and once again showed capacity to reliably exceed 1 under slow SOA conditions. Simultaneous EEG recording during Experiment 4 revealed the neural signatures associated with the encoding and retrieval phases of the paradigm. In particular, it appears the repeated failure of AV capacity to exceed 1 under 200 ms SOA condition is due to the inability of the visual cortex to successfully code the number of changing locations in frames prior to the critical one. Experiment 5 complements the main experimental series by demonstrating similarly uninformative locational cues presented in the visual modality did not yield large estimates of *K* [after 1], supporting the claim that the paradigm assesses the capacity of audio-visual integration. We conclude with an across-experiment comparison of modelled *K* estimates and proportion data, and a discussion that reinforces the idea that audio-visual capacity can be dynamic.

## Experiment 1

### Method

#### Participants

We received informed, written consent from 24 participants prior to experimentation. All participants were recruited from an undergraduate research participant pool, and were compensated with partial class credit. The Research Ethics Board at Ryerson University approved the experimental procedure and recruitment practices, in accordance with the Declaration of Helsinki. Before data analysis, we calculated a 95% confidence interval around 50% (chance responding) over 384 trials. We then removed the data of any participant who was performing within the 95% CI on average across all 8 conditions. 6 individuals were rejected on the basis of chance responding, resulting in a final sample of 18 participants– 2 males and 16 females—with a mean age of 19.7 years (SD = 4.8), and a total of 16 right handed individuals. All participants self-reported normal or corrected-to-normal vision and hearing.

#### Stimuli

Visual stimuli were presented on a Viewsonic VE175 monitor at a screen resolution of 1280 x 1024 and a refresh rate of 60 Hz, using an ACPI PC running Windows 7 Professional (Service Pack 1) at a viewing distance of approximately 57 cm. Auditory stimuli were presented binaurally via Sennheiser HD 202 headphones. Stimulus presentation was controlled by Presentation (NBS, 2013) version 16.5, build 09.17.13, and behavioural responding was recorded with a Dell SK-8165 keyboard. Dots (1.3° in diameter) could be displayed in one of two colours: black (0, 0, 0) or white (255, 255, 255) against a mid-grey background (128, 128, 128). The main phase of the experiment consisted of the simultaneous display of eight dots along an implied circle (13° in diameter), the center of which was marked by a 1.5° fixation dot. A single, smaller probe dot was overlaid on a target dot at the end of each trial, and was red (255, 0, 0) with a diameter of 1°. The auditory stimulus used was a 60 ms long, 400 Hz tone with 5 ms linear on-set and off-set ramps, presented at an intensity of approximately 74 dB(C), which was created using SoundEdit 16 (MacroMedia).

#### Design and procedure

48 individual conditions of stimuli were created, by orthogonally varying the SOA of visual stimuli (200 ms, 700 ms), the number of visual stimuli that changed on each alternation (1, 2, 3, 4), the critical frame (7th, 8th, 9th), and the validity of the probe stimulus (valid, invalid). These 48 conditions were each presented once to create an experimental block. Each participant completed one practice block, and 8 experimental blocks, for a total of 384 experimental trials. Trial order was randomized in practice and in experimental trials.

**Fig 1:**
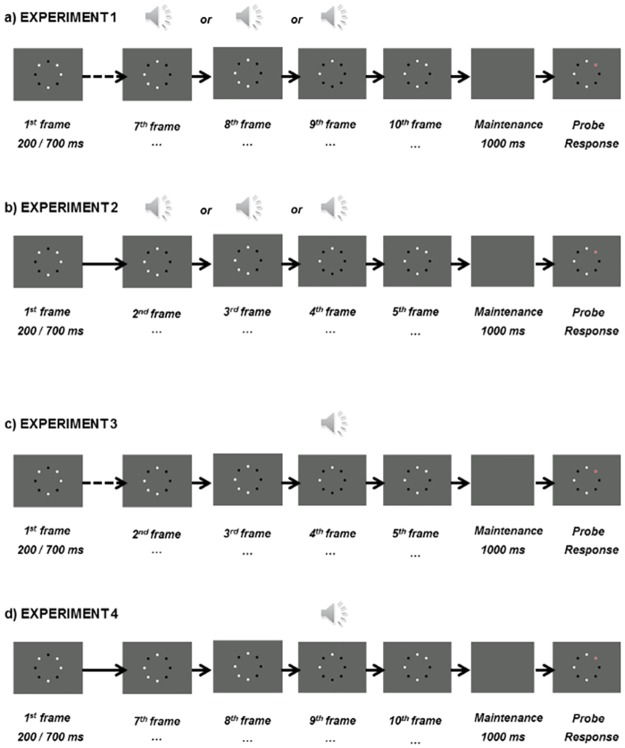
Schematic representation of the procedures in Experiments 1–4. Panels a) to d) correspond to each of Experiments 1 to 4.

Fig 1a provides a schematic of the critical aspects of Experiment 1. Each trial began with a fixation point displayed in the center of the screen for 500ms. The sets of black and white dots were generated independently for each trial, and there was no restriction on which dot(s) could change colour at each alternation, nor was there a restriction on how many dots could be white or black at any one time. The first array of dots was presented for either 200 or 700 ms (dependent on condition), and subsequent arrays followed immediately at SOAs of 200 or 700 ms.

On the 7th, 8th, or 9th frame, the onset of the dots was accompanied by an auditory tone. Following the final (10th) presentation, a 1000 ms retention interval occurred during which only the fixation point was displayed on the screen. During the retrieval phase, the tenth array of dots was then displayed again, with the same locations black and white as were when it was first presented, along with an overlay of a red probe dot on one of the eight dots. Participants were asked to respond to whether the dot at the probe location had changed or not on the critical display by pressing the number 1 on the number pad if that dot did not change, and by pressing the number 2 on the number pad if that dot did change. Response buttons were not counterbalanced. The probe had a validity of 50%, and the location for invalid trials was randomly determined. No feedback was provided, and the subsequent trial began immediately after a response was entered.

#### Model Fitting

Data were modelled in the same manner as employed by [1]. The proportion correct for each condition and for each participant was fitted to a model equivalent to Cowan’s *K* [3], wherein if n ≤ *K*, then *p* = 1, and when n > *K*, then *p* = *K*/2n + .5 (n represents the number of visual elements changing (1–4), and *K* is an estimate of capacity of audio-visual binding). That is to say, if a participant’s capacity was equal to or greater than the number of locations changing on a given trial, their probability of correct responding was 1. However, if the number of locations changing exceeded individual capacity, errors would occur and the capacity estimate would reflect this based on the specific number of locations changing. Trials were collapsed across critical frame and validity. Data were fitted to this model by using Microsoft Excel Solver, and fitting was initiated from several starting values of *K*. The fitting procedure performs an optimization procedure, fitting to a curve with the lowest root mean square error (RMSE) for each SOA. The outcome with the smallest RMSE was selected, and this process was done independently for each participant and SOA condition.

### Results and Discussion

**Fig 2:**
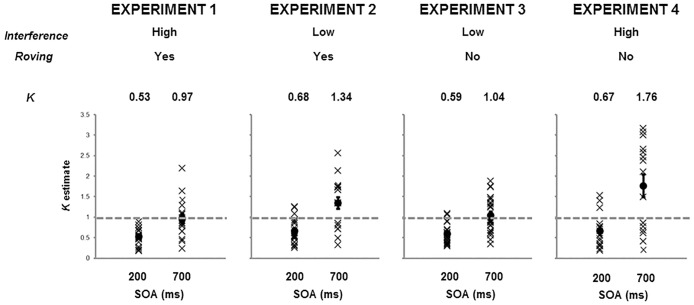
Graph to show variability of audio-visual integration capacity (*K*) for each of Experiments 1–4. Individual participant data points are indicated with an X, condition means are indicated with a dot. Error bars depict standard error.

Successful model fit was confirmed by the low RMSEs observed in both the 200 ms SOA (range .001 –.056) and 700 ms SOA (range .001 –.059) conditions, consistent with the RMSE range of .036 - .060 reported in [1] (p. 347). *K* values for each SOA were compared with the proposed capacity of 1 by means of single sample t-tests (see Fig 2a). Average *K* value in the 200 ms condition was significantly less than 1 (0.529 [range 0.180–0.885]; *t*(17) = -9.36, *p* < .001) and the average *K* value in the 700 ms condition was not significantly different from 1 (0.967 [range 0.241–2.187]; *t*(17) = -0.29, *p* = .772). Capacity at 200 and 700 ms SOAs were compared by means of a paired-samples t-test, revealing a significant advantage for 700 ms (*t*(17) = 4.58, *p* < .001). The group data from Experiment 1 are consistent with the idea that the capacity of audio-visual integration cannot exceed 1 [1], although the plot of individual data clearly shows some individuals whose capacity was greater than 1.

## Experiment 2

One essential design feature associated with the original Van der Burg et al. [1] paradigm is that there are a number of polarity-changing frames presented prior to the critical audio-visual binding frame. Successful performance on the task relies on participants tracking location changes throughout the trial, so that they are able to discriminate which location changed on the critical frame. However, it is well established that when previously perceived information remains in working memory, it inhibits the perception of future stimuli (*proactive interference*; [14]) and also interferes with participants’ ability to successfully allocate attention [15]. This effect has been shown to be stronger when using abstract visual stimuli (such as the black and white dots used here) relative to higher level stimuli such as words or pictures [16]. In this respect, it is possible that the preceding frames proactively interfere with the frame of interest, decreasing performance and hence the capacity of audio-visual integration. In Experiment 2, we attempted to increase audio-visual integration capacity by reducing the number of frames preceding the audio-visual frame (and hence the amount of proactive interference). In Experiment 2 from an initial sample of 24 participants, a total of 5 participants were rejected based on their response criterion, so that the final sample consisted of 19 participants– 14 females—with a mean age of 23.4 years (SD = 6.7), and a total of 17 right handed individuals. None of the participants had taken part in Experiment 1 and all self-reported normal or corrected-to-normal vision and hearing. The design of Experiment 2 was identical to Experiment 1, apart from the pre-maintenance phase now containing 5 frames, with the auditory signal (critical frame) being presented on the 2nd, 3rd or 4th frame (see Fig 1b).

### Results and Discussion

Successful model fit was confirmed by the low RMSEs observed in both the 200 ms SOA (range .003 –.030) and 700 ms SOA (range .002 –.039) conditions.

For the 200 ms condition, *K* was significantly less than 1 (0.658 [range 0.261–1.258]; *t*(18) = -4.85, *p* < .001) while for the 700 ms condition *K* was significantly greater than 1 (1.342 [range 0.317–2.562]; *t*(18) = 2.49, *p* = .023; see Fig 2b). A paired-sample t-test showed that 700 ms capacity was significantly greater than 200 ms (*t*(18) = 7.33, *p* < .001). The observation of an estimate of audio-visual integration capacity significantly greater than 1 following a reduction in proactive interference is in direct contradiction of the claim of Van der Burg et al. [1] but is not without precedent. A recent paper [17] also reported that a single auditory cue can influence the nature of the interaction between two *pairs* of moving dots in terms of a stream / bounce judgement, at least when one of the pairs is presented simultaneously with the auditory cue and the other pair is presented separately in time, but within the *temporal window of integration* of the sound (e.g., [18]). Given that in both our slow and fast SOA conditions, all visual changes on the critical frame were presented simultaneously with the auditory cue, our data also align with the idea that for multiple visual events to be associated with a single auditory cue, they must be presented within a critical temporal integration period.

## Experiment 3

In both Experiments 1 and 2, the critical frame was not temporally predictable across trials and we predict this further added to the difficulty of the task. To take a uni-modal example, [19] used fixed or variable trial lengths in testing the perception of a light appearing at the end of the trial and found that performance decreased in the variable trial length condition. Therefore, in the hope of improving task performance and AV capacity further in Experiment 3, the critical frame was fixed on the fourth frame (out of five; see Fig 1c). 1 participant was rejected on the basis of response criterion so that the final sample consisted of 23 participants– 2 males and 21 females—with a mean age of 19.6 years (SD = 2.5), and a total of 21 right handed individuals. All participants reported normal or corrected-to-normal hearing and vision, and no participant had taken part in Experiments 1 or 2.

### Results and Discussion

Successful model fit was confirmed by the low RMSEs observed in both the 200 ms SOA (range .003 –.053) and 700 ms SOA (range .001 –.059) conditions.

The capacity for the 200 ms condition was significantly less than 1 (0.590 [range 0.296–1.093]; *t*(22) = -8.94, *p* < .001) and the capacity for the 700 ms condition was not significantly greater than 1 (1.042 [range 0.341–1.880]; *t*(22) = 0.44, *p* = .661; see Fig 2c). Capacity for 700 ms was significantly higher than for 200 ms, as indicated by a paired-samples t-test (*t*(22) = 6.39, *p* < .001) The data from Experiment 3 were surprising in that the combination of two factors that should have improved AV capacity (reduced proactive interference with temporal predictability) did not. This raises the possibility that the task in Experiment 3 may have been too easy, failing to generate the requisite level of arousal for participants to stay on task. Conversely, Experiment 1 (proactive interference with temporal roving) may have been too difficult to support the appropriate allocation of attention to the various visual and auditory components [15]. In contrast, Experiment 2 may have generated an intermediate level of task difficulty by combining the lack of temporal predictability (thereby increasing difficulty) with a low degree of proactive interference (thereby reducing difficulty). We further test the idea of intermediate task difficulty in Experiment 4 by eliminating temporal roving but maintaining a relatively high level of proactive interference. Additionally, we also captured neural activity by simultaneously recording EEG to test a critical assumption of the paradigm.

## Experiment 4

Successful performance on the current variant of the pip-and-pop paradigm [1] relies on identifying which visual locations changed in polarity when an auditory cue is presented. Logically, one can only have a sense of which locations *changed* at any one frame by successfully registering the status of the various locations during preceding frames. Failure to discriminate between the number of changing locations (1, 2, 3, 4) in the frames leading up to the critical one would suggest that participants do not have the prerequisite perceptual information required to successfully identify which location(s) changed at the time the auditory cue was presented. Consistent in this regard is a previous report by Van der Burg et al. [20], who show that with fast rates of presentation between 50 and 250 ms, basic exogenous responses such as P1 and N1 fail to generate in visual cortex. In Experiment 4, we test the hypothesis that the AV capacity of 1 observed at fast SOA is due to poor quality sensory (visual) information entering working memory by examining neural responses at both encoding and retrieval phases of the paradigm.

During encoding of the non-critical frames, the neural component that could be most reliably compared between a trial running at 200 ms SOA and 700 ms SOA is the visual N1. Given that the visual N1 is sensitive to a number of characteristics including the magnitude of physical change, attentional allocation and the eventual requirement of a discriminatory response ([21], [22]), we believed the N1 should index the ability of the visual cortex to discriminate between the number of to-be-tracked locations: a neural prerequisite of the task would be the coding of location change numerosity, confirming that the visual cortex is sensitive to the number of locations that change polarity at each trial.

During the retrieval phase in which participants responded to the probe, we also anticipated the observation of posterior N2 and P3b components as indices of visual selection (e.g., [23]–[25]) and the initiation of a response resulting from perceptual analysis (e.g., [26]). Consistent with the data in [20] we assumed that the components should be maximal at posterior sites, thereby locating one site of AV integration at posterior inferior cortex. In particular, the magnitude of N2 should increase as the number of successfully individuated visual objects increases [23] and P3b should be reflective of the degree to which the location is behaviourally relevant [20]. Finally, by examining the correlation between neural response at encoding and retrieval phases with behavioural response, it was possible to ascertain the degree to which successful eventual performance was associated with perceptual processes (encoding) and / or post-perceptual processes (retrieval).

### Method

From an initial sample of 25 participants prior to experimentation, a total of 9 participants were rejected due to a biased response criterion. An additional 3 participants were rejected because of low quality EEG recording. As such, the final behavioral sample consisted of 16 participants– 3 males and 13 females—with a mean age of 18.5 years (SD = 1.1), and a total of 15 right handed individuals. The final EEG sample consisted of 13 participants– 3 males and 10 females—with a mean age of 18.5 years (SD = 1.2), and a total of 13 right handed individuals. Although the EEG sample size is relative small, it is consistent with the *n* = 14 reported in [20]. All participants self-reported normal or corrected-to-normal vision and hearing and had not taken part in any previous experiments. Experiment 4 was identical to all previous experiments, apart from the unique combination of standard level of proactive interference (from Experiment 1) and the absence of temporal roving (from Experiment 3).

#### Electrophysiological recording

Electrical brain activity was continuously digitized using Acti-View (Bio-Semi; Wilmington, NC), with a band-pass filter of 208 Hz and a 1024 Hz sampling rate. Recordings made from FPz, F3, Fz, F4, C3, Cz, C4, PO7, P3, Pz, P4, PO8, T7, T8, POz, Oz, M1, M2, CMS and DRL were stored for off-line analysis. Horizontal and vertical eye movements were recorded using channels placed at the outer canthi and at inferior orbits, respectively. Data processing was conducted using BESA 5.3 Research (MEGIS; Gräfelfing, Germany). Following average referencing, the contributions of both vertical and horizontal eye movements were reduced from the EEG record, using the VEOG and HEOG artefact options in BESA [27]. Using a 0.1 (12 db/oct; zero phase) Hz high-pass and 30 (24 db/oct; zero phase) Hz low-pass filter, epochs were rejected on the basis of amplitude difference exceeding 100 μV, gradient between consecutive time points exceeding 75 μV, or, signal lower than 0.01 μV, within any channel. Following average mastoid re-referencing (to maintain parity with [1]), neural activity was averaged across posterior sites PO7, POz, PO8, P3, Pz and P4 electrode sites. For the encoding phase, the first eight non-critical frames in the 200 ms SOA condition were baseline corrected 100 ms prior stimulus onset and activity was examined 200 ms following stimulus onset. In the 700 ms SOA condition, baseline correction was established 200 ms prior to the stimulus and activity was examined for the first eight non-critical frames 700 ms following the stimulus. The retrieval stage of audio-visual integration (probe response presentation; see Fig 1) was defined according to a baseline of 200 ms prior to stage and 1000 ms following the onset of the stage. The total number of epochs analyzed per participant is shown in S1 File, and summarized in S1 Table.

### Results

Successful model fit for the behavioural data was confirmed by the low RMSEs observed in both the 200 ms SOA (range .001 –.028) and 700 ms SOA (range .002 –.036) conditions. In the 200 ms SOA condition, the capacity was found to be significantly less than 1 (.667 [range 0.180–1.525]; *t*(14) = -3.03, *p* = .009), while in the 700 ms SOA condition the capacity was found to be significantly greater than 1 (1.763 [range 0.205–3.160]; *t*(14) = 2.74, *p* = .016; see Fig 2d). A paired-sample t-test indicated that the capacity at 700 ms was significantly greater than at 200 ms (*t*(14) = 5.42, *p* < .001). Similar to the data from Experiment 2, the data from Experiment 4 confirm that the capacity of audio-visual integration can once again exceed 1.

**Fig 3:**
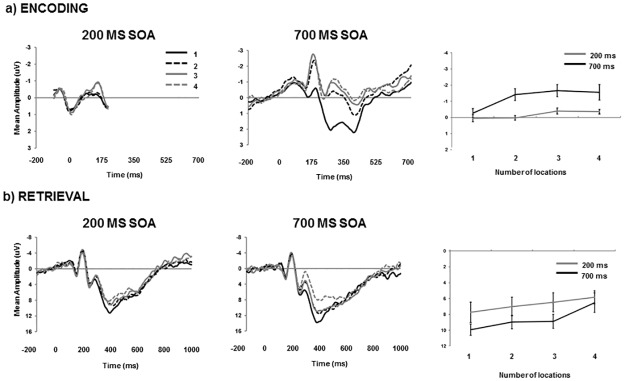
**(a) Mean waveforms for each SOA and number of objects changing during the encoding phase (first 8 non-critical frames) in Experiment 4.** Line graph depicts N1 mean amplitude. **(b) Mean waveforms for each SOA and number of objects changing during the retrieval phase (probe response frame) in Experiment 4.** Line graph depicts N2 and P3b mean amplitude. Error bars depict standard error.

Fig 3a presents aggregated neural activity at each of the two stages of audio-visual integration (encoding, retrieval) as a function of SOA and the number of to-be-tracked visual locations. Directly comparing the visual N1 (100–200 ms) response at the time of encoding between the two SOAs reveals significant main effects of presentation rate (F[1,12] = 9.39, *p* = .010, ƞ_p_^2^ = .439), number of locations (F[3,36] = 9.49, *p* < .001, ƞ_p_^2^ = .442) and an interaction (F[3,36] = 3.37, *p* = .029, ƞ_p_^2^ = .219). The interaction in the left panel of Fig 3b demonstrates a lack of sensitivity to the number of visual locations in the 200 ms SOA condition. This is in contrast with the reduction of the visual N1 when participants tracked only one location using the 700 ms SOA relative to the other conditions (all Tukey’s HSD; *p* < .05). In order to compare the relative effects of number of location and SOA more clearly, paired sample t-tests were used to compare N1 amplitude at 200 and 700 ms SOA at each number of locations changing. Due to multiple comparisons being conducted, an adjusted critical *p* value of .00625 was used in determining significance. This comparison was not significant when one (*t*(12) = .733, *p* = .477) or four (*t*(12) = 2.479, *p* = .029) locations were changing, but was significant when two (*t*(12) = 3.815, *p* = .002) or three (*t*(12) = 3.369, *p* = .006), locations were changing. This indicates that SOA played a role in the level of degradation of visual stimuli only when there was an intermediate number of locations to be tracked.

At the time of retrieval, N2 and P3b responded in a similar fashion to the number of locations allowing for their joint analysis. Neural activity incorporating both N2 and P3b (250–600 ms) revealed decreased positivity as a function of fast relative to slow SOA: (F[1,12] = 7.45, *p* = .018, ƞ_p_^2^ = .382) and decreased positivity generated by an increase in the number of visual locations that were tracked (F[3,36] = 10.08, *p* < .001, ƞ_p_^2^ = .457). Specifically, 4 locations produced significantly less positivity than all other conditions (Tukey’s HSD; *p* < .05). The interaction term was not significant (F[3,36] = 0.75, *p* = .529, ƞ_p_^2^ = .059).

**Fig 4:**
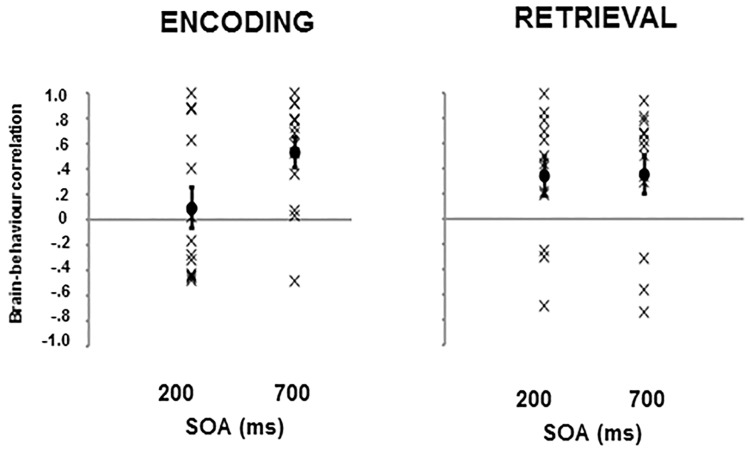
Correlation between ERP mean amplitude and behavioural data during encoding and retrieval stages, as a function of SOA. Individual participants’ correlations are indicated with an X, condition means are indicated with a dot. Error bars depict standard error.

More specific analyses of N2 (235–285 ms) and P3b (335–385 ms) mean amplitude revealed main effects of SOA ((F[1,12] = 17.44, *p* = .001, ƞ_p_^2^ = .592) and (F[1,12] = 17.68, *p* = .001, ƞ_p_^2^ = .596), respectively) and location ((F[3,36] = 9.08, *p* < .001, ƞ_p_^2^ = .430), and, (F[3,36] = 18.95, *p* < .001, ƞ_p_^2^ = .612), respectively), in addition to two-way interactions between SOA and location ((F[3,36] = 4.07, *p* = .014, ƞ_p_^2^ = .253), and, (F[3,36] = 4.32, *p* = .011, ƞ_p_^2^ = .264), respectively). The interaction showed that at the earlier stages (235–385 ms) of the broader epoch (250–600 ms) reported above, the decreased positivity observed as a function of the reduction in visual locations was more pronounced during the slow SOA condition. Fig 4 summarizes the relationship between brain and behavior at encoding and retrieval stages of audio-visual integration according to SOA. Correlations were calculated by examining mean amplitude and proportion correct associated with the 4 levels of visual location, for each participant separately for encoding and retrieval phases (see [28], for a similar procedure). Average correlations were compared against zero in a series of one-sampled t-tests. The data reveal the significance of encoding (*r* = .530; *t*(12) = 4.42, *p* < .001) and retrieval (*r* = .351; *t*(12) = 2.62, *p* = .039) during 700 ms SOA presentation, and the significance of the retrieval stage (*r* = .340; *t*(12) = 2.45, *p* = .031) but not encoding (*r* = .093; *t*(12) = 0.58, *p* = .574) during 200 ms SOA presentation.

### Discussion

Using a second expression of intermediate task difficulty in Experiment 4 (no temporal roving but a high degree of proactive interference), we observed the average capacity of audiovisual integration rising above 1 during slow rates of presentation (see also Experiment 2). This is in contrast to the estimate of AV capacity during fast presentation rates, which again appeared fixed around 1. The origin of this behavioural difference appears to stem from differential neural activity between the two SOA conditions during the encoding rather than the retrieval phase. As previously stated, in order for participants to know which location(s) change on the critical frame they must have some sense of how many (and which) locations changed on frames preceding the critical one. In other words, cortical sensitivity to location change numerosity would appear to be a prerequisite for successful performance in the task.

What our data show is that there is no reliable distinction in the visual cortex to the number of locations changing during pre-critical frames under conditions of fast (200 ms) SOA. If the visual cortex has not correctly registered the number of changing locations, it also seems unlikely that the specific coordinates of these changes would be reliably registered. In these respects, the previously reported ‘limit’ of AV capacity could be an epiphenomenon of fast stimulus presentation ([12], [13]) leading to an indiscriminate response in visual cortex [20]. Thus, the quality of perceptual information entering working memory also plays a role in defining the capacity of audio-visual integration. In contrast, brain-behaviour correlations for fast and slow presentation rates were equivalent during the retrieval phase, where participants were probed as to the validity of one specific location. Consistent with previous data [20], [23], [28], posterior N2 decreased and P3b increased as the number of tracked locations decreased.

Whether the lack of reliable cortical sensitivity to different location changes at 200 ms SOA represents a real data-limit to the ultimate capacity of audio-visual integration could be tested in future experiments that utilize a passive viewing condition. Although while the contents of the display were directly task-relevant in the current design, it would be of interest to see whether- upon passive viewing of the same displays- differential N1 activity could be observed across the various locational change conditions. One possibility is that having to keep track of *which* locations changed actually inhibited the registration of *how many* locations were actually changing (in some form of dual-task decrement). If N1 activity successfully distinguished between the number of locations during passive viewing at a rate of 200 ms SOA, then it would suggest that poorer performance during fast SOAs were not the result simply of a fixed data-limit but rather a more complex interaction between bottom-up and top-down processes. The extent to which 200 ms SOA represents a true data-limit could have been further explored by the additional recording of ERP in Experiments 1–3. If overall task difficulty interacts with the capacity of AV integration at relatively slower (but not relatively faster) SOAs, then we might expect to see a continued lack of visual N1 for location change number irrespective of overall task difficulty in 200 ms SOA conditions but significant modulation of visual N1 in 700 ms SOA conditions, according to whether the task was overall easy, intermediate or hard, relative to our current manipulations of temporal predictability and proactive interference.

## Cross-Experiment Analysis of *K* Estimate Data

**Fig 5:**
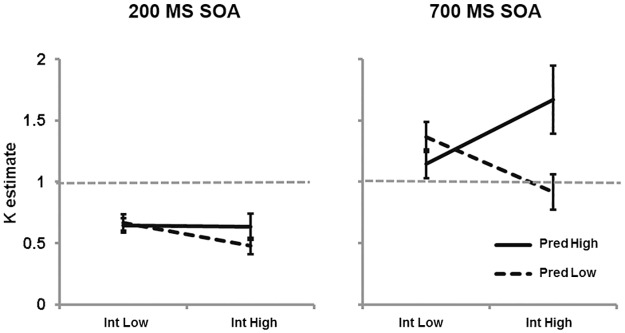
Graph to show estimates of audio-visual integration capacity (*K*) as a function of SOA, interference and temporal predictability. Error bars depict standard error.

In order to consolidate our understanding of how the capacity of audio-visual integration modulates as a function of SOA, proactive interference and temporal roving, *K* estimates across Experiments 1–4 were entered into a three-way mixed-model ANOVA including the between-participants factor proactive interference (low, high), the between-participants factor temporal roving (no, yes) and the within-participants factor SOA (200, 700). Full ANOVA results are presented in Table 1 and graphical presentation of the means are presented in Fig 5. A main effect of SOA (*p* < .001) confirmed that estimates of K were significantly larger during 700 ms SOA (1.279) than 200 ms SOA (0.611). Interactions between interference x roving (*p* = .003) and a three-way interaction (*p* < .001) were further explored by conducting separate two-way analyses for 200 ms and 700 ms SOA conditions. For the faster rate of presentation (200 ms) there were no main effects or interaction (all *p*s > .077). In contrast, the slower rate of presentation (700 ms) showed an interaction between interference x roving (*p* = .002), revealing significance between more interference + no roving (1.763; Experiment 4) and more interference + roving (0.968; Experiment 1).

**Table 1:** Summary of the three-way mixed model ANOVA comparing estimates of *K* across Experiments 1–4 (all *df*s 1,71).

Metric	F	MSE	*p*	*ƞ*_*p*_^*2*^
**Rove (R)**	1.82	.402	.182	.025
**Interference (I)**	0.49	.402	.486	.007
**SOA (S)**	**137.07**	**.119**	**< .001**	.659***
**R x I**	**9.66**	**.402**	**.003**	.120*
**R x S**	3.46	.119	.067	.046
**I x S**	3.07	.119	.084	.041
**R x I x S**	**15.23**	**.119**	**< .001**	.177**

Statistical significance indicated by bold text.

* = small effect size (*η*_*p*_^*2*^ > .02)

** = medium effect size (*η*_*p*_^*2*^ > .13)

*** = large effect size (*η*_*p*_^*2*^ > .26)

The data are instructive in confirming that performance during 200 ms SOA was insensitive to experimental manipulation. While this would suggest the impenetrability of audio-visual integration capacity during fast presentation, the data from the 700 ms SOA condition clearly show capacity modulation and estimates that exceed 1. Therefore, the data underscore the importance for future research to consider task difficulty as a large scale influence of AV capacity, indexed by a relatively complex interaction between SOA, the degree of proactive interference, temporal roving of the critical frame, and perceptual load, to name but 4 factors. Other than slow SOA, cases in the current experimental series do not point to one particular aspect of the design that gives rise to flexible AV capacity. For example, Experiment 2 (700 ms SOA; *K* = 1.342) combined reduced proactive interference and the lack of critical frame temporal predictability, whereas Experiment 4 (700 ms SOA; *K* = 1.763) combined increased proactive interference with temporal predictability. For the moment we simply suggest that these specific environmental designs represent intermediate levels of task difficulty allowing for the modulation of AV capacity. Such cases stand in contrast to paradigms employing fast rates of presentation, temporal roving of the critical frame, and large degrees of proactive interference, which tend to generate conservative estimates of audio-visual integration capacity.

## Cross-Experimental Analysis of Proportion Correct Data

While the main interest of this experimental series was to examine the factors that influence the capacity of audio-visual integration, it was also of interest to examine the raw proportion of correct responses. This analysis was instructive as to when the differences between SOA conditions manifest themselves. It was expected that, in general, proportion correct would be lower in the 200 ms SOA as compared to the 700 ms SOA, and that it would decrease with an increase in number of locations to be tracked. More specifically, as the task became of increasing difficulty (e.g. more locations to be tracked) we expected there to be an increase in the facilitative effect of other factors, such as temporal predictability and proactive interference.

**Fig 6:**
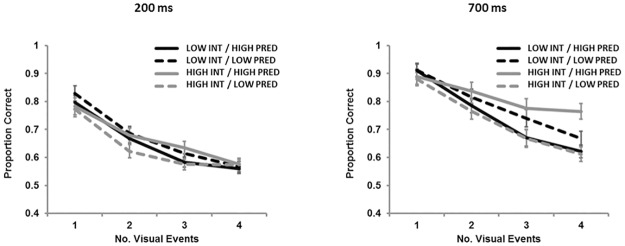
Proportion correct for each combination of temporal predictability, proactive interference, and number of locations changing for 200 ms SOA (left panel) and 700 ms SOA (right panel) for Experiments 1–4. Error bars indicate standard error.

The proportion correct data (shown in Fig 6) from all 4 experiments were submitted to a mixed ANOVA with between-subjects factors of Interference (2: high, low) and Roving (2: high predictability, low predictability), and within-subjects factors of SOA (2: 200 ms, 700 ms) and Number of locations to be tracked (4: 1, 2, 3, 4), which is summarized in Table 2. There was a main effect of SOA (*p* < .001), with improved responding in the 700 ms condition than in the 200 ms condition. There was also a main effect of Number (*p* < .001), with each additional item to be tracked decreasing performance significantly. A trend towards an SOA x Number interaction (*p* = .061) revealed that while in the 700 ms condition there were incremental decreases in performance with added items to be tracked, in the 200 ms condition this was only true up to 3 items, with the fourth item not having added decrement beyond the third. This follows well with the account of a sensory barrier, and with the finding that there was no modulation of K in the 200 ms condition while there was in the 700 ms condition.

**Table 2:** Summary of the four-way mixed model ANOVA comparing raw proportion correct data across Experiments 1–4 (all *df*s 1,71; apart from main effects and interactions with events [E] *df*s 3,213).

Metric	F	MSE	*p*	*η*_*p*_^*2*^
**Rove (R)**	0.60	.053	.441	.008
**Interference (I)**	0.01	.053	.936	.001
**SOA (S)**	**195.19**	**.009**	**< .001**	.733***
**Experiment (E)**	**252.83**	**.006**	**< .001**	.781***
**R x I**	**5.39**	**.053**	**.023**	.071*
**R x S**	1.41	.009	.239	.019
**R x E**	0.97	.006	.406	.014
**I x S**	1.44	.009	.234	.020
**I x E**	**4.19**	**.006**	**.007**	.056*
**S x E**	2.37	.005	.072	032
**R x I x S**	**4.39**	**.009**	**.040**	.058*
**R x I x E**	**3.12**	**.006**	**.027**	.042*
**R x S x E**	1.15	.005	.331	.016
**I x S x E**	0.25	.005	.860	.004
**R x I x S x E**	**4.19**	**.005**	**.007**	.056*

Statistical significance indicated by bold text.

* = small effect size (*η*_*p*_^*2*^ > .02)

*** = large effect size (*η*_*p*_^*2*^ > .26)

A significant Interference x Number interaction (*p* = .001) showed that in high and low interference conditions there were different effects stemming from the number of items to be tracked. While there was also no difference between interference conditions at any number of items to be tracked, performance was marginally better under conditions of no proactive interference when the number of tracked events was small (1 or 2) but that performance was marginally better under conditions of proactive interference when the number of tracked events was large (4; consistent with the observations of [29]), but none of these pairwise comparisons were statistically significant.

Finally, there was a 4-way interaction between Interference x Roving x SOA x Number (*p* = .007). This interaction was probed by means of Tukey’s (*p* < .05) post-hoc tests, and analyses were focused on the relationships between conditions that were present within a specific number of items to be tracked. When there was one item to be tracked, we saw SOA effects at every combination of interference and roving, as well as a difference between the high interference, low temporal predictability, 200 ms condition and both low interference 700 ms condition points. We also see SOA effects at each interference/roving combination in the 2-change and 3-change conditions, and only in the low interference/low predictability and high interference/high predictability conditions in the 4-change condition. Beyond SOA effects, we see an effect of temporal predictability in the 2-change, high-interference condition, as well as in the 3-change, high-interference condition. To generalize these findings, it can be said that intermediate difficulty levels lead to the highest levels of integration capacity. Specifically, only in these intermediate difficulty conditions does SOA have an impact on response accuracy when four locations are changing—the hardest level of that factor—and therefore it can be concluded that it is under those conditions that capacity could be maximized.

## Experiment 5

A potential criticism of the current experimental series is that performance on the 700 ms SOA condition is attainable not only through audio-visual integration, but also through visual cueing (Van der Burg, personal communication, 1 May 2015). In order to provide evidence against this alternative explanation, an additional experiment was conducted in which the auditory cue at the critical stimulus was replaced with a visual cue. If participants are able to perform at an equivalent level using a visual cue relative to an auditory cue, then our assumptions regarding the previous data reflecting estimates of audio-visual integration can rightly be questioned. However, if participants are unable to perform at similar levels using a similarly locationally uninformative *visual* cue, then this will provide evidence that audio-visual integration is occurring at both 200 and 700 ms SOAs in our previous experiments.

### Method

Following [1; Experiment 1d], in Experiment 5 the experimental parameters were identical to Experiment 4 apart from the use of a non-specific visual cue (rather than non-specific auditory cue) at the time of the critical frame. The visual cue consisted of the presentation of two concentric green circles (RGB; 0, 128, 0) positioned 1 degree inside and outside of the ring of dots. 12 participants were recruited and compensated with $10 cash. The mean age was 28.3 (SD = 4.2), with 9 females and 12 right handed individuals. Attempts to exclude participants based on near-chance responding using the procedure in Experiment 1–4 would have eliminated 8 of the 12 participants, so no participants were excluded for the purposes of this control experiment.

### Results and Discussion

Fitting was performed in the same way as in Experiments 1–4, with RMSE indicating a good fit (.001 - .043 for 200 ms; .004 - .043 for 700 ms). As in the previous experiments, *K* values were compared to a test value of 1 using a single sample t-test. These t-tests indicated that capacity was significantly less than 1 for both 200 ms (*K* = 0.12; *t*(11) = -38.680, *p* < .001) and 700 ms (*K* = 0.23; *t*(11) = -12.195, *p* < .001) conditions. A paired sample t-test showed that there was no significant difference between capacity at 200 and 700 ms (*t*(11) = 1.793, *p* = .101). The capacity estimate and range from the 200 ms SOA condition in Experiment 5 (0.12; range 0.01–0.24) also map on to the findings reported by [1; Experiment 1d] (0.13; range 0–0.36). Critically, performance at both 200 ms and 700 ms SOA were significantly different between Experiments 4 and 5, as a mixed ANOVA showed that Experiment 4 had a greater capacity than Experiment 5 (F(1, 25) = 43.164, MSE = 25.692, *p* < .001), indicating that the mechanisms in binding visual cues to vision are not the same as the mechanisms in binding auditory cue to vision.

The potential limitations of these findings should be considered, given that the concentric circles being used as a visual cue could serve to mask the critical stimulus change. If attention were to be drawn away from the discs at the moment of stimulus change, the visual stimulus could serve as a distractor rather than a cue. However, use of this particular methodological variation is taken directly from the research of [1], and was used in their research as evidence that a visual cue is not sufficient to lead to the same type of integration as seen in audiovisual stimulus conditions. As such, the findings from Experiment 5 provide support to the contention that we are examining audio-visual integration in Experiments 1–4.

## General Discussion

Across Experiments 1–4, we established some conditions under which the capacity of audio-visual integration may exceed 1. Specifically, visual set size should be low [11] and stimulus change should operate at a slow rather than fast rate of presentation [12]. Capacity can go beyond 1 when there is temporal roving and low proactive interference (Experiment 2) or no temporal roving with high proactive interference (Experiment 4). Neither of these contributions predict increased AV capacity in isolation and so it is likely that intermediate task difficulty provides the appropriate levels of arousal for successful performance ([30]; and for a similar example in the context of multitasking see [31]). The single paradigm feature that does seem to be necessary for high AV capacity is the use of relatively slow (700 ms) compared to relatively fast (200 ms) SOA. While the impenetrability of audio-visual integration at 200 ms SOA might suggest some form of limit, the possibility remains that this is a data limit rather than capacity limit [2]. This is supported by our electrophysiological data and particularly our analysis of visual N1 during pre-critical frame presentations. Here, visual cortex was insensitive to the number of polarity changes per frame in the 200 ms SOA condition but sensitive to similar changes during the 700 ms SOA condition. Here, we take the indiscriminate brain response during fast rates of presentation to reflect poor quality sensory information entering working memory, and the failure of the brain to complete an initial tracking task that is a prerequisite for successful performance in the task. Future research should seek to unpick the fidelity of sensory representation when neural responses to individual stimuli overlap [32, 33], as was the likely case in the 200 ms SOA condition.

It is probably also worth restating that despite their claim, in the original Experiment 1c of [1], their range of *K* estimates was 0.70–1.56, indicating that some of their individuals also exceeded 1 (see also their Experiment 2, discussed below). We replicate the observation that certain individuals expressed capacity beyond 1, to 2 and even 3 (see Fig 2). To defend the position that ‘the capacity of audio-visual integration is limited to one item’ when there is data that some participants, at least on some of the trials, were able to bind two visual locations (or more) to a single auditory source appears contradictory. At the very least, the ranges cited above raise the clear need to further study individual difference in audio-visual capacity, much in the same way as it has received attention in the context of VSTM (e.g., [34]).

We discuss three potential objections to the current data. First, the data of Van der Burg et al. ([1]; Experiment 2) provide surface evidence against the idea that the reason why AV capacity cannot exceed 1 under 200 ms SOA conditions is due to the inability to successfully code the number of changing locations in frames prior to the critical one. Here, they show that under visual-only conditions running at an SOA comparable to ours (150 ms), *K* was estimated around 3.34, whereas in an audio-visual condition presented at the same fast speed, *K* comes in around 0.78 (range = 0.30–1.36). A primary reading of the data would suggest that capacity was 3 when the task was visual-only, but capacity could not exceed 1 when the task was audio-visual. This apparently shows then under visual-only conditions, participants can track, on average, at least 3 locations. However, the comparison between the visual-only and audio-visual conditions is not an appropriate one. Specifically, the signal for the critical frame in the visual condition was marked by a unique color change (from white/black to green) at *specific dot locations*. The use of such a salient colour cue is likely to have given rise to perceptual pop-out at target locations during the critical frame, additionally meaning that it would have been unnecessary for participants to track location changes in frames prior to the critical one. We believe these effects yield the high *K* in the visual condition and do not support the idea that multiple locations can be tracked during particularly fast rates of presentation. Moreover, the lack of cueing to specific target locations in the auditory condition in their Experiment 2 undoubtedly contributes to the observation of lower *K* in that condition. We also note that in the same paper, their previous Experiment 1d, which we replicated and extended in our Experiment 5, uses a non-location specific visual cue which was more comparable with the auditory case, by the authors own admission: “a cue that, like the sound cue, was not specific to any of the items” ([1], p. 349). Under these conditions in which the comparison between visual-only and audio-visual performance was more valid (both location non-specific), performance in the visual-only condition was poor (*K* = .56).

Second, there may remain opposition to idea that audio-visual integration capacity may exceed 1 since it only apparent during a slow (700 ms) rate of presentation. There are a number of responses to this, foremost the lack of evidence suggesting that qualitative changes in audio-visual binding should arise as the result of the manipulation of a continuous variable such as SOA: in contrast to the claim that 700 ms is ‘too slow’ to allow for integration across the senses there is evidence that neurons in the superior colliculus are sensitive to audio–visual integration at an asynchrony of 600 ms ([35], cited in [36]). Furthermore, it is not clear that faster rates of presentation (such as 150 and 200 ms) provide an error-free index of AV capacity. [1] notes that slowing the rate of presentation from 150 ms to 200 ms SOA improved AV capacity probably as a result of “the reduced likelihood of misbindings” between auditory and visual events ([1], p. 348). Given this significant increase in performance as a result of slowing SOA by 50 ms, there can be little surprise that audio-visual capacity exceeds 1 with further extension.

Furthermore, we move away from the possibility that the capacity of audio-visual integration is stuck at 1 due to spuriously poor performance caused by a high degree of illusory audio-visual binding. It is possible to empirically test the idea of illusory binding in future studies and we plan to do so. Given the temporal preference for auditory-lag rather than auditory-lead in binding sound with vision (e.g., [37]) one prediction would be that under fast rates of presentation, participants incorrectly bind to the preceding rather than current visual frame. Therefore, there should be an increased number of responses that are ‘incorrect’ in accordance with the critical frame but ‘correct’ in accordance with the frame preceding it. Objections to slow delivery rates would also appear to confuse trial SOA with the importance of the degree of temporal separation between visual and auditory event at the critical frame. That is, during the critical frame, both visual and auditory on-sets occur simultaneously (0 ms difference), falling within the typical temporal window of integration required for audition and vision (e.g., [18]). As noted by [17], the delivery of auditory and visual events within a shared window of integration appears to be an essential characteristic if one wishes to associate a single auditory event to multiple visual events. Finally, if the mechanisms underlying performance in the present task qualitatively change at 700 ms SOA, then we would expect an exponential increase in capacity at 700 ms SOA, relative to the increases observed between 200 ms SOA and an intermediate 450 ms SOA. Initial data from these new investigations we are currently conducting suggest this is not the case and furthermore we observe AV capacity greater than 1 at a group level following training on an intermediate 450 ms SOA.

A third and final objection to excessive audio-visual integration may originate in an appeal to ecological validity, and the observation that in the real world unique sounds tend to have a single (visual) source. To wit: “From an ecological point of view, it would make sense to bind only one visual event to a specific sound. In natural scenes, individual, object-related sounds (unlike the sound of the wind or a babbling brook) come from a single source…” ([1], p. 345–346). Leaving aside the examples in the above quote, one might imagine a relatively large-scale auditory scene, such as an orchestra, where multiple visual events (e.g., violin section) give rise to a specific, streaming sound. In terms of the larger context of audiovisual integration, the data are particularly important as they show that the capacity of integration can exceed one when temporal and stimulus factors cannot adjudicate between visual candidates that may be bound to the auditory event: all changes occur at the same point in time and involved polarity shifts of the same nature. When both temporal and stimulus factors are completely ambiguous with respect to audio-visual integration, it seems entirely plausible that the system should attempt to bind all of the visual stimuli with the auditory stimulus as source *candidates*–with the possibility that some post-hoc information may later emerge that might disambiguate them. For the moment, the current data support the contention that the capacity of audio-visual integration is a dynamic process and reveal the environmental conditions and concomitant brain states under which it need not be limited to 1.

## Supporting Information

S1 File Data for Experiment 1–5. (XLSX)

S2 File Total number of epochs per participant entered into encoding and retrieval analyses as a function of SOA (200, 700) and number of locations changed (1, 2, 3, 4). (XLSX)

S1 Table Average number of epochs entered into encoding and retrieval analyses as a function of SOA (200, 700) and number of locations changed (1, 2, 3, 4). (DOCX)
