# Supplementary material for: The Dynamics and Neural Correlates of Audio-Visual Integration Capacity as Determined by Temporal Unpredictability, Proactive Interference, and SOA
Source: PLoS One. 2016 Dec 15;11(12):e0168304. doi: 10.1371/journal.pone.0168304 (PMC5158043; doi:10.1371/journal.pone.0168304)
Supplement: S1 Table — (DOCX) [file pone.0168304.s003.docx]

Table S1

Average number of epochs entered into encoding and retrieval analyses as a function of SOA (200, 700) and number of locations changed (1, 2, 3, 4)

_____________________________________________________________________________________________________________

SOA 200 ms 700 ms

LOCATION 1 2 3 4 1 2 3 4

_____________________________________________________________________________________________________________

PHASE

Encoding 358 (28) 358 (33) 359 (31) 360 (33) 326 (46) 327 (42) 326 (44) 329 (45)

Retrieval 29 (6) 24 (7) 24 (6) 21 (5) 31 (6) 30 (9) 26 (8) 27 (7)

_____________________________________________________________________________________________________________

Note: Standard deviation supplied in parentheses.
